# Structure of the Bacteriophage PhiKZ non-virion RNA Polymerase transcribing from its promoter p119L

**DOI:** 10.1016/j.jmb.2024.168713

**Published:** 2024-07-17

**Authors:** Natàlia de Martín Garrido, Chao-Sheng Chen, Kailash Ramlaul, Christopher H. S. Aylett, Maria Yakunina

**Affiliations:** 1 Section for Structural and Synthetic Biology, Department of Infectious Disease, Imperial College London, London, United Kingdom; 2 Shenzhen MSU-BIT University, 1 International University Park Road, Dayun New Town, Longgang District, Shenzhen, Guangdong Province, People’s Republic of China, 518172

**Keywords:** β subunit, RNA polymerase, β′ subunit, Cryo-EM, Jumbo-phage, σ-factor, Single-particle analysis, phiKZ

## Abstract

Bacteriophage ΦKZ (phiKZ) is the founding member of a family of giant bacterial viruses. It has potential as a therapeutic as its host, *Pseudomonas aeruginosa*, kills tens of thousands of people worldwide each year. ΦKZ infection is independent of the host transcriptional apparatus; the virus forms a “nucleus”, producing a proteinaceous barrier around the ΦKZ genome that excludes the host immune systems. It expresses its own non-canonical multi-subunit non-virion RNA polymerase (nvRNAP), which is imported into its “nucleus” to transcribe viral genes. The ΦKZ nvRNAP is formed by four polypeptides representing homologues of the eubacterial β/β’ subunits, and a fifth that is likely to have evolved from an ancestral homologue to σ-factor. We have resolved the structure of the ΦKZ nvRNAP initiating transcription from its cognate promoter, p119L, including previously disordered domains and regions. Our results shed light on the similarities and differences between ΦKZ nvRNAP mechanisms of transcription and those of canonical eubacterial RNAPs and the related non-canonical nvRNAP of bacteriophage AR9.

## Introduction

Bacteriophage ΦKZ is the prototypical member of a family of bacterial viruses distantly related to the Myoviridae (Krylov et al., 2021; Krylov et al., 2007; Krylov & Zhazykov, 1978). It infects *Pseudomonas aeruginosa*, which is an intrinsically antibiotic-resistant opportunistic pathogen of special concern in medical practice as it kills tens of thousands of people annually (Stover et al., 2000). As bacteriophage ΦKZ can effectively combat *Pseudomonas aeruginosa* infection, it has been considered a potential candidate for bacteriophage therapy (Hall et al., 2012; Med Sci et al., 2018; Pires et al., 2015). ΦKZ was the first bacteriophage classified as “giant” due to its exceptional size, encapsulating a huge, densely packed DNA genome of 280 334 bp (Mesyanzhinov et al., 2002). Bacteriophage ΦKZ has a highly divergent life cycle and its massive genome encodes many proteins that would usually be expected to be provided by the host (Prichard et al., 2023). To protect the ΦKZ genome from host immune systems it forms a membrane vesicle in the early stages of infection (Antonova, Nichiporenko et al., 2023; Armbruster et al., 2023) and a proteinaceous “nucleus” within the host cytoplasm during the later stages (Chaikeeratisak, Nguyen, Egan et al., 2017; Danilova et al., 2020; Mendoza et al., 2020). To manage the intracellular traffic during ΦKZ infection a tubulin cytoskeleton based on bacteriophage proteins is constructed inside the cell (Aylett et al., 2013; Chaikeeratisak et al., 2019; Chaikeeratisak, Nguyen, Egan et al., 2017; Chaikeeratisak, Nguyen, Khanna, et al., 2017).

The lifecycle of ΦKZ in *P. aeruginosa* is unaffected by the host RNA polymerase (RNAP) inhibitor rifampicin, indicating that ΦKZ is independent of the host transcription machinery. While most bacteriophage-encoded polymerases are single-subunit RNAPs (ssRNAPs), ΦKZ is one of the few bacteriophages that encodes two non-canonical multi-subunit RNAPs (msRNAPs) (Campbell et al., 2001; Ceyssens et al., 2014; Sokolova et al., 2020): a virion RNAP (vRNAP) that is injected with the bacteriophage genome during infection to transcribe early genes, and a non-virion RNAP (nvRNAP) responsible for transcription of all further genes needed for the assembly of new bacteriophages to complete the lytic cycle (Clark et al., 1974; Sokolova et al., 2017; Yakunina et al., 2015). In the case of bacteriophage ΦKZ this independent transcriptional strategy may be favoured due to the isolation of bacteriophage genome from host transcriptional machinery at all stages firstly by placing it into the membrane vesicle and secondly by enveloping it within the bacteriophage nucleus (Antonova, Nichiporenko et al., 2023), whereas in other cases of related non-canonical msRNAPs, such as bacteriophage AR9, the genome contains uracil in place of thymine, and this base is required for transcription initiation (Sokolova et al., 2017).

Canonical msRNAPs exhibit a conserved double-ψ β-barrel (DPBB) domain in two large, related subunits, β and β’ in bacteria (Forrest, 2018; Iyer et al., 2003; Lane & Darst, 2010a; Sokolova et al., 2020), however the β/β’ heterodimer is catalytically inactive without addition of a dimer of α subunits (Heyduk et al., 1996; Minakhin et al., 2001; Zaychikov et al., 1996; Zhang et al., 1999). Although this core is catalytically active, exchangeable σ-subunits are required to initiate transcription from cognate promoters (Lane & Darst, 2010a; Lonetto et al., 1992). ΦKZ encodes two sets of genes that are homologous to the two largest subunits of bacterial msRNAPs, β and β’ (Ceyssens et al., 2014), but does not possess homologues of the α and ω subunits required for the assembly of a catalytically active enzyme in bacterial msRNAPs (Heyduk et al., 1996; Minakhin et al., 2001). The ΦKZ vRNAP is formed by at least five polypeptides (GP178, GP149, GP180, GP80 and GP176), whereas the ΦKZ nvRNAP is formed by four subunits (GP55, GP71-73, GP74 and GP123) which are homologous to the two largest subunits of bacterial msRNAPs (β and β’), and a fifth, GP68, with no detectable sequence homology to any other protein which is responsible for promoter recognition and transcription initiation (Yakunina et al., 2015).

We previously determined the structure of the ΦKZ nvRNAP (de Martín Garrido, Orekhova, et al., 2021), revealing that the architecture of the ΦKZ nvRNAP resembles that of eubacterial msRNAPs, and the four β/β’-like subunits retain most of the conserved structural elements needed for the reaction cycle and stabilisation of the transcription bubble. The fifth subunit, GP68, is found at the same location as bacterial σ factors, and conserves some of the structural elements of these factors, suggesting that GP68 has probably evolved from a common ancestor with bacterial σ factors. Important parts of the polymerase such as domains of GP68, GP74 and GP123 remained unresolved, however.

The structure of the related AR9 promoter complex has also been resolved bound to a 3’-overhang dsDNA template mimicking one half of the transcription bubble, without any RNA present (Fraser et al., 2022). This structure revealed a related set of split β/β’-like subunits and σ factor-like subunit, as well as an uracil-specific template-strand mechanism of promoter recognition not applicable to bacteriophage ΦKZ as this base is not present in its DNA. Unfortunately, the ΦKZ nvRNAP promoter is not well-defined: its consensus is extremely short (4-5 bp depending on how it is determined) and overlaps the initiation site, while transcription initiation is not well-behaved *in vitro*. The roles of sequences both further up, and downstream from the transcription initiation site have yet to be determined in detail, however there are no clear consensus sequences in either direction.

In this study we have resolved the cryo-EM structure of the ΦKZ nvRNAP bound to an RNA/DNA template transcribing from its promoter p119L to a resolution of 3.3 Å, uncovering the architecture of the domains that were not resolved in the ΦKZ nvRNAP holoenzyme structure (de Martín Garrido, Orekhova, et al., 2021). Our results provide further insights into the role of the intriguing fifth subunit, GP68, allowing comparison with the related bacteriophage AR9 nvRNAP, and revealing structural details of the mechanism of transcription by non-canonical msRNAPs of giant bacteriophages.

## Results and Discussion

### Generation of ΦKZ nvRNAP-p119L/RNA complexes suitable for cryo-EM studies

**Figure 1:**
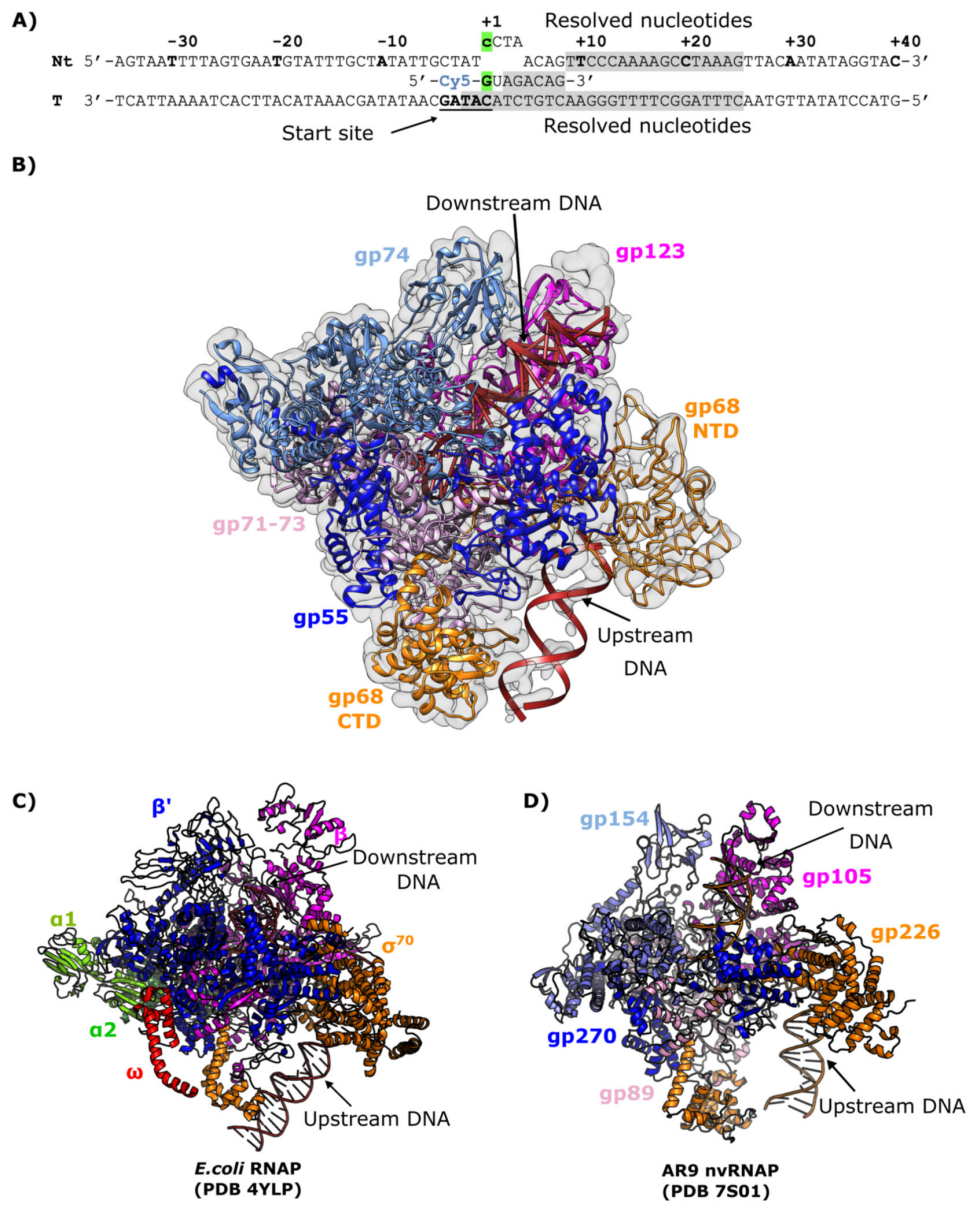
Overview and architectural elements of ΦKZ nvRNAP transcribing p119L in a halted post-translocation state. (A) Primary structural representation of the p119L – Cy5-RNA transcription template. The PhiKZ late promoter 4-nucleotide consensus sequence overlapping with the transcription start “C”(+1) marked in bold underlined letters “ATAC” on the template strand. In the non-template strand, the sequence inside (+1: +4) was modified to create an artificial transcription bubble. The grey regions represent the sections of nucleotide sequence sufficiently well-resolved to allow modelling *de novo*. The green boxes show the +1 nucleotide position on the RNA and non-template strand. (B) Structure of ΦKZ nvRNAP transcribing p119L with a locally filtered cryoEM map prepared with LAFTER (Ramlaul *et al*., 2019). The β-like subunits GP71-73 and GP123 (or equivalent regions in AR9 / *E. coli* structures) are coloured pink and magenta respectively; β’-like subunits GP74 and GP55 (or equivalents) are coloured light and dark blue respectively; σ-like subunit GP68 (or equivalent) is coloured orange; α-subunits are shown in red, ω-subunits in green; template DNA is coloured black, non-template DNA is in white, and RNA is in silver. (C) Structure of *E. coli* RNAP transcription initiation complex (PDB 4YLP) (Zuo & Steitz, 2015) for comparison. (D) Structure of the AR9 nvRNAP (PDB 7S01) (Fraser et al., 2022) for comparison.

We expressed and purified recombinant ΦKZ nvRNAP complexes from *Escherichia coli* using a co-expression system with plasmids bearing the four β/β’-like proteins and GP68 as in our previous study. We set out to obtain a transcribing complex of the ΦKZ nvRNAP, a strategy that has been successful in obtaining cryo-EM structures for other polymerases (Glyde et al., 2018; Liu et al., 2017). For this purpose, we designed a DNA template covering positions -35 to +40 of the specific ΦKZ late promoter p119L, containing a 4-nucleotide artificial bubble directly after nucleotide +1, and a 5-nucleotide RNA scaffold at the transcription start site (Figure 1A; Supp. Figure 1). A transcription reaction was performed without UTP in order to halt transcription at the first adenine encountered by the polymerase within the template strand. Under these conditions, the nvRNAP would be expected to transcribe three nucleotides (up to position +8). For structure determination, ΦKZ nvRNAP-p119L/RNA complexes were stabilised by adsorption to a graphene oxide film covering a holey carbon copper grid and vitrified before visualisation by cryo-EM.

### Reconstruction and generation of an atomic model of the ΦKZ nvRNAP-p119L/RNA complex

We recorded images of well-populated fields of particles and subjected them to single particle analysis (Supp. Figure 2). Extensive particle sorting and focused refinement allowed us to recover a well-resolved reconstruction of the ΦKZ nvRNAP-p119L/RNA complex to a final resolution of 3.3 Å (Independent half-set FSC = 0.143) (Supp. Figures 3-4). From initial comparison of our prior ΦKZ nvRNAP apo-structure (de Martín Garrido, Orekhova, et al., 2021) with the new reconstruction we could clearly observe extra regions of density (Figure 1B; Supp. Figure 5). Comparison with an *E. coli* RNAP transcribing complex (PDB 6GH5) (Glyde et al., 2018), suggested that the new 3D reconstruction exhibited weakly resolved DNA entering and exiting the ΦKZ nvRNAP channel in a similar manner to that in bacterial msRNAPs (Figure 1C). Moreover, this new reconstruction allowed us to build an atomic model covering almost all of the four β/β’-like subunits (GP71-73, GP123, GP55 and GP74) encompassing 97% of their sequence, and to dock an Alphafold2 structural model covering the N-terminus of GP68 (Figure 1B; Supp. Figure 5). Furthermore, our structure included 6-bp of the RNA transcript along the template strand at the catalytic core, as well as the downstream dsDNA accommodated within the polymerase channel. The upstream dsDNA was not resolved to high enough local resolution to assign its sequence, however we were able to dock a dsDNA model tracing the phosphate backbone. As we had previously found in the ΦKZ nvRNAP apo-structure, the overall shape of the transcribing nvRNAP resembled that of bacterial msRNAPs and most of the structural elements involved in the transcription reaction were observed to be conserved (de Martín Garrido, Orekhova, et al., 2021). Accordingly, the DNA entering and exiting the nvRNAP channel was positioned in a similar manner to that in eubacterial msRNAPs (Figures 1C-1D).

### The ΦKZ nvRNAP catalytic core is conserved in structure and function from eubacterial msRNAPs

**Figure 2:**
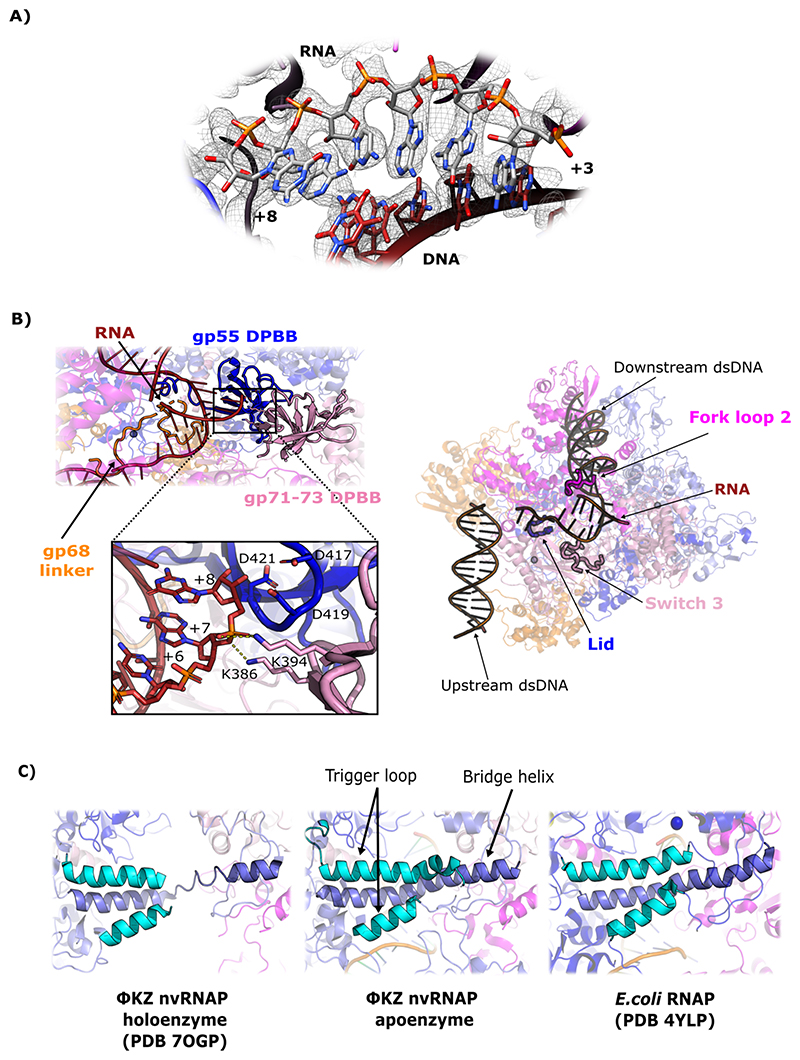
The catalytic centre of the ΦKZ nvRNAP transcribing p119L in a halted post-translocation state. (A) Inset of the catalytic centre of the ΦKZ nvRNAP. RNA is represented in grey and template DNA in firebrick red, whereas the electron density is represented in a grey mesh. (B) The catalytic core of the ΦKZ nvRNAP; each subunit is depicted with a different colour: gp123 in magenta, gp71-73 in pink, gp55 in blue, gp74 in light blue and gp68 in orange. The two DPBBs are contributed by GP55 and GP71-73 and the GP68 linker invades the catalytic clef. Inset shows the aspartate triad (417-DFDGD-421) contacting the last residue transcribed (+8) and the two universally conserved lysines (K386 and K394) contact the RNA transcript in the upstream nucleotides. (C) Inset showing the ΦKZ nvRNAP trigger loop and bridge helix in the holo (left) and p119L (centre), and *E. coli* (right) structures.

At the catalytic centre of the ΦKZ nvRNAP structure initiating transcription from the p119L RNA/DNA template, we observed clear density for 6-bp of the 8-bp RNA transcript hybridised along the single-stranded DNA template strand (Figure 2A). However, the non-template single-stranded DNA forming the transcription bubble was not visible in our map. As we observed in the ΦKZ nvRNAP apo-structure, the catalytic cleft was formed by the two conserved double-Ψ-β-barrel (DPBB) domains found in GP55 and GP71-73. The universally conserved motif 417-DFDGD-421 was included in GP55 forming a similar loop to that found in *E. coli* transcribing complexes. The three aspartates contained in this motif coordinate the first catalytic Mg^2+^ needed for the nucleotide addition reaction. The three aspartates are located as if the Mg^2+^ would be contacting the recently added nucleotide (3’), however the ion was not clearly distinguishable at the resolution of our map. The other DPBB found in GP71-73 contained two positively charged lysines (K386, K394) that interacted with the nascent RNA (Figure 2B). Overall, our observations provide further experimental evidence to suggest that the eubacterial transcription mechanism and catalysis of RNA polymerisation are most likely conserved in the nvRNAPs of giant bacteriophages.

### The ΦKZ nvRNAP-p119L/RNA complex retained key DNA/RNA stabilisation elements in conformations representing a halted post-translocation state of transcription

**Figure 3:**
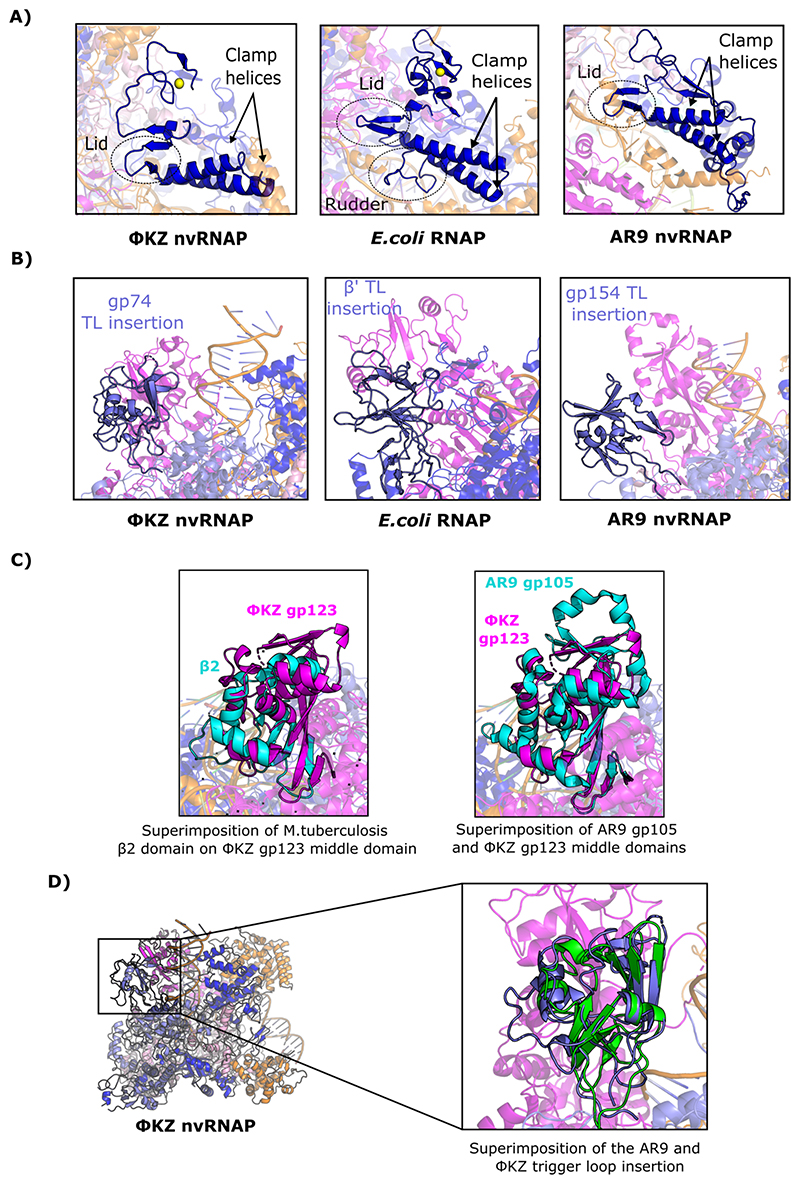
Newly resolved ΦKZ nvRNAP-p119L features are intermediate between eubacterial and AR9 msRNAPs. (A) Structural comparison of the ΦKZ nvRNAP and *E.coli* RNAP structural elements included in the clamp; (left) ΦKZ nvRNAP p119L/RNA complex showing the structural elements of the clamp and the Zn^2+^ binding domain at the N-terminus of GP55, (centre) *E. coli* RNAP transcribing complex (PDB 4YLP) (Zuo & Steitz, 2015) showing the structural elements of the clamp, (right) AR9 nvRNAP promoter complex (Fraser et al., 2022) showing the structural elements of the clamp. Note the rudder is absent in ΦKZ, and both rudder and the Zn^2+^ binding domain are absent in AR9. (B) Comparison of the ΦKZ nvRNAP (left) with the *E. coli* (centre) and AR9 (right) RNAP trigger loop insertions; the inset shows the interaction between the Arg508 of GP74 and the phosphate backbone at +16 relative to the transcription start site. (C) The ΦKZ GP123 corresponds to the β2 lobe in *M. tuberculosis* (PDB 6JCY) (Fang et al., 2019) (superimposition in centre), and AR9 (superimposition in right); the superimposed structure is shown in cyan in each panel. (D) Superimposition of the AR9 and the ΦKZ trigger loop insertion. All panels are coloured identically. The β-like subunits GP71-73 and GP123 (or equivalent regions in AR9 / *E. coli* structures) are coloured pink and magenta respectively; β’-like subunits GP74 and GP55 (or equivalents) are coloured light and dark blue respectively; σ-like subunit GP68 (or equivalent) is coloured orange; α-subunits are shown in red, ω-subunits in green; template DNA is coloured black, non-template DNA is in white, and RNA is in silver.

We observed that many of the structural elements of the holoenzyme which are key for transcription had different conformations in the ΦKZ nvRNAP structure transcribing from the p119L RNA/DNA template. The trigger loop was fully visible in the ΦKZ nvRNAP-p119L/RNA structure (Figure 2C), displaying a helix/loop conformation, and the bridge helix was in a fully helical conformation, in comparison to the nvRNAP structure without DNA (de Martín Garrido, Orekhova, et al., 2021). The clamp is a mobile element that oscillates between different conformations throughout the transcription reaction, adopting a closed conformation that stabilises the DNA during the RNAP promoter open complex (Chakraborty et al., 2012). In the ΦKZ nvRNAP structure bound to RNA/DNA template, we observed that the clamp was fully ordered with the two clamp helices and the lid adopting similar conformations to those found in bacterial msRNAP transcribing complexes (Figure 3A). The lid interacted with the flap to enclose the RNA exit channel in the same way as in bacterial msRNAPs. In eubacterial msRNAPs, fork loop 2 and switch 3 of the β subunit, as well as the lid and rudder included in the β’ subunit, form a network of interactions with the DNA and RNA strands to stabilise the transcription bubble. Therefore, most of the structural elements at the catalytic centre of the ΦKZ nvRNAP involved in transcription bubble formation are similar to those in bacterial msRNAPs (Figures 2A-2C). These observations suggest that the DNA is probably loaded into the ΦKZ nvRNAP in a similar manner to eubacterial msRNAPs, using universally conserved structural elements found in the split β/β’-like subunits, and implies that features of the elongation cycle are also conserved from canonical msRNAPs.

### The ΦKZ nvRNAP clamp may represent an evolutionary situation between the states previously observed in eubacterial and AR9 msRNAPs, retaining a Zn binding domain but without the β’-rudder

The majority of the ΦKZ nvRNAP DNA/RNA stabilisation elements of the channel are conserved from eubacterial msRNAPs as we have reported above. Notably, however, we found that the rudder of the β’ subunit was absent in the homologous ΦKZ nvRNAP subunit GP55 (Figure 3A). The rudder is typically found within the clamp and is involved in the stabilisation of the unwound DNA in the transcription bubble of elongation complexes (Kuznedelov et al., 2002), suggesting that other parts of the enzyme are likely to be involved in this function. The rudder is also a key transcriptional feature missing from the only non-canonical msRNAP homologue of the ΦKZ nvRNAP resolved, the nvRNAP of bacteriophage AR9 (Fraser et al., 2022). At the N-terminus of GP55, close to the clamp, we found a Zn^2+^ binding domain formed by four cysteine residues coordinating the ion in a similar manner than in the *E.Coli* RNAP (Figure 3A). A zinc binding domain at the N-terminus of the β’ subunit is a shared feature of all msRNAPs (Lane & Darst, 2010a; 2010b). While this structural element is conserved in the ΦKZ nvRNAP, it is not observed the nvRNAP of AR9 (Fraser et al., 2022) (Figure 3A; Supp. Figure 6). This situation suggests that the ΦKZ nvRNAP may be placed between the eubacterial and AR9 msRNAPs evolutionarily and may imply that promoter opening by the ΦKZ nvRNAP is closer to the canonical pathway than the uracil-dependent mechanism of the AR9 nvRNAP.

### The GP123 insertion was found to correspond to the β2-lobe of eubacterial msRNAPs, and is likely to perform the same role in transcription

One of the largest regions of the ΦKZ nvRNAP structure that had been disordered within our original apo-structure was the GP123 middle domain (146-336). This region was substantially better-resolved in the ΦKZ nvRNAP-p119L/RNA structure. We performed a structural comparison of the GP123 β2 domain using the DALI server and found that the closest structural homologue was the β subunit of *M. tuberculosis* (RMSD 3.1 Å according to DALI), although GP123 had additional insertions (Figure 3C). Moreover this part of GP123 corresponded to the bacterial β2 lobe, which covers the downstream dsDNA in eubacterial msRNAPs (Lane & Darst, 2010b) and indeed, the GP123 middle-domain forms the tip of one of the pincers that holds the downstream dsDNA in our structure. Although this region was not resolved to high resolution in our map, GP123 appears to contact the DNA through a loop that is partially disordered in our map and is rich in positively charged residues (192-TKRDLGN-198).

### The trigger-loop insertion domain within GP74 is unrelated to that in *E. coli* msRNAP, having a similar fold to that found in the AR9 nvRNAP

The other large domain that was newly resolved in the ΦKZ nvRNAP structure bound to the p119L RNA/DNA template was found within GP74. This domain is an insertion within the trigger loop (396-533). The *E. coli* RNAP also has a lineage-specific insertion in the trigger loop, comprising residues 942-1129 of the β’ subunit, which is involved in different stages of transcription (Sutherland & Murakami, 2018). We observed that the ΦKZ nvRNAP trigger loop insertion in GP74 had a different fold to that of the equivalent *E. coli* insertion (Figure 3B). We found that the trigger loop insertion of the ΦKZ nvRNAP was located next to the GP123 middle domain, enclosing the downstream dsDNA (Figure 3B). The AR9 subunit homologous to the C-terminus of the bacterial β’ subunit also contains an insertion after the trigger loop (referred to as the TL domain in (Fraser et al., 2022)), similar to that observed for the ΦKZ subunit GP74. Superimposition of the two trigger loop insertion domains shows that they have a nearly identical fold (C_α_ RMSD 2.4 Å), with most secondary structure elements being found in the same places (Figure 3D). We also found that the GP74 insertion was in a different position within the complex to that in the AR9 structure, probably because both structures were obtained using DNA templates mimicking different transcription intermediates, and the trigger loops are therefore in different conformations.

### GP68 contacts upstream DNA through its CTD in a manner different from the situations in both AR9 GP226 and eubacterial σ factor

**Figure 4:**
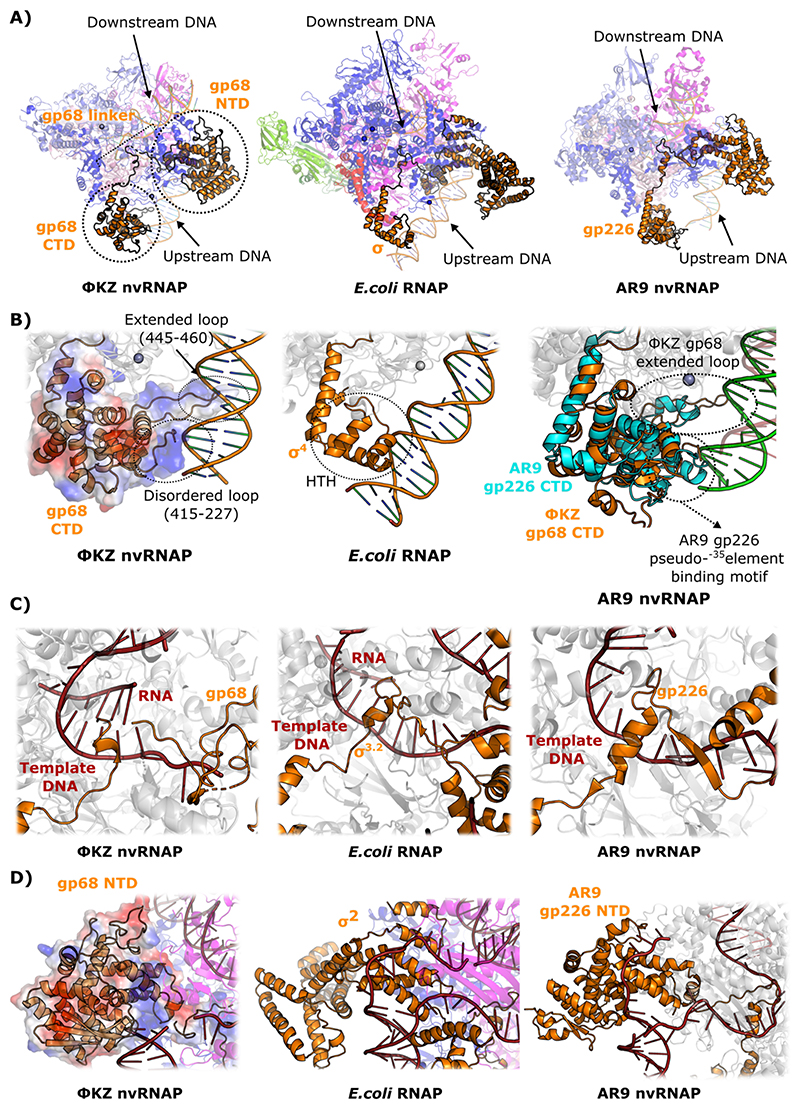
Comparison of ΦKZ nvRNAP-p119L GP68 with the promoter complex of AR9 nvRNAP supports a permanently attached but otherwise σ^E^-like mechanism of transcription initiation. (A) The GP68 architecture and position is similar to bacterial σ-factors and the AR9 promoter specificity factor. (left) ΦKZ nvRNAP p119L/RNA, (centre) *E. coli* RNAP transcription initiation complex (PDB 4YLP) (Zuo & Steitz, 2015), (right) AR9 promoter (Fraser et al., 2022), with σ-factor highlighted in each case. (B) Structural comparison of the GP68 CTD (left), bacterial σ^70^ region 4 (centre) and AR9 CTD (right); the electrostatic potential is depicted as a transparent surface with blue representing positive charge regions, white neutral, and red negative regions; black dotted lines encircle putative DNA binding interfaces. (C) Structural comparison of the GP68 (left) and AR9 (right) linkers with bacterial σ^70^ region 3 (centre). (D) Structural comparison of the GP68 (left) and AR9 (right) NTDs with bacterial σ^70^ region 2 (centre); electrostatic potential is represented as a transparent surface with blue regions representing positive charges, red representing negative and white neutral. All panels are coloured identically, and in each case the regions of interest have been coloured to highlight them, whereas other sections are represented in translucent greys. The β-like subunits GP71-73 and GP123 (or equivalent regions in AR9 / *E. coli* structures) are coloured pink and magenta respectively; β’-like subunits GP74 and GP55 (or equivalents) are coloured light and dark blue respectively; σ-like subunit GP68 (or equivalent) is coloured orange; α-subunits are shown in red, ω-subunits in green; template DNA is coloured black, non-template DNA is in white, and RNA is in silver.

Bacteria usually encode a principal σ factor responsible for transcribing most of the constitutive genes and also contain alternative σ factors which transcribe genes involved in specific functions, such as virulence-associated genes or those involved in stress response (Browning & Busby, 2004; Paget & Helmann, 2003). In eubacteria, transcription initiation requires the binding of a σ factor to specifically initiate transcription from promoters (Saecker et al., 2011). Our previous study implied that the fifth subunit GP68, essential for promoter recognition, is embedded within the complex, making extensive contacts with GP71-73 and GP55, rather than a temporary member, and probably evolved from an ancestral σ factor (de Martín Garrido, Orekhova, et al., 2021). As we previously observed in the ΦKZ nvRNAP apo-structure, the GP68 CTD corresponds to bacterial σ region 4 (de Martín Garrido, Orekhova, et al., 2021). Region 4 of the bacterial σ factor is a mostly helical domain that interacts with the conserved -35 element of bacterial promoters through a helix-turn-helix motif (Campbell et al., 2002; Murakami et al., 2002). The GP68 CTD exhibits the same architecture with and without nucleic acids and was in a position suitable to interact with the upstream DNA through two different interfaces. The first contact involved a disordered loop (415-427) that was placed in a similar position within the complex to the helix-turn-helix motif of σ factor (Figure 4A). The second putative GP68-DNA contact involved an extended loop (445-460), which would contact the DNA in a more downstream region through its tip. The electrostatic potential and the amino acid composition of both the disordered loop and the extended loop (which are rich in positively charged and hydrophobic amino acids), supports a role of these two GP68 CTD regions in DNA binding (Figure 4B). Due to the limited resolution of our map in this region, we could not assign sequence to the upstream DNA, hence assignment of specific promoter regions involved in interactions with the GP68 CTD was not possible. Although the percentage of sequence similarity between them was 18%, the promoter recognition subunit of AR9, GP226, had the same structural organisation as that of the ΦKZ GP68: two helical domains, the NTD and CTD, connected by a linker (Fraser et al., 2022). The CTD of the AR9 nvRNAP (GP226) was found to be structurally similar (C_α_ RMSD 3.651 Å when residues 262-461 of GP226 were superimposed on residues 274-521 of GP68). Both domains were located in the equivalent position of region 4 of bacterial σ factor, however both the bacteriophage subunits were expanded as previously observed (de Martín Garrido, Orekhova, et al., 2021). The CTD of AR9 GP226 contacted the DNA at approximately region -35 through a loop (386-395) (Fraser et al., 2022) in contrast to region 4 of bacterial σ factors that utilizes a helix-turn-helix motif (Campbell et al., 2002; Murakami et al., 2002). The authors termed this CTD GP226 region the pseudo--35-element-binding motif (Fraser et al., 2022), and the equivalent region in ΦKZ GP68 corresponds to the first potential upstream interaction we note above (415-427) (Figure 4B). Unlike bacterial promoters, which have a conserved -35 element, neither AR9 promoters nor ΦKZ promoters recognised by their respective nvRNAPs show sequence conservation in regions interacting with the promoter-recognition CTD, which suggests that these interactions are likely to be novel and sequence independent. The second putative interaction of the gp68 CTD with DNA, which we have previously termed the extended loop, is not present in the bacteriophage AR9 nvRNAP. Notably, it was shown that the upstream region is required for efficient transcription from the p119L promoter by the ΦKZ nvRNAP (Yakunina et al., 2015).

### The GP68 linker recapitulates features of eubacterial σ factor, and therefore the mechanism of promoter release appears likely to be conserved

During elongation, when RNA transcripts reaches 12-15 nucleotides in length, and σ factor dissociates from the msRNAP core (ββ’α_2_ω) (Mooney et al., 2005). The mechanism by which this occurs in eubacterial msRNAPs is displacement of the σ^3.2^ loop when transcripts reach a certain length in order to unblock the RNA exit channel (Murakami et al., 2002). The GP68 subunit is formed by two domains, the CTD (314-521) and the NTD (1-272) which are physically separated by a linker (273-313), meaning that the overall structure of GP68 and its position within the complex resemble that of bacterial σ factors (Figure 4A). Resolving the structure of the ΦKZ nvRNAP complex bound to a specific p119L RNA/DNA template allowed us to partially model the GP68 linker. At the active centre of the nvRNAP, we observed that the GP68 linker invaded the catalytic cleft in a similar manner to the σ^3.2^ loop. The tip of the GP68 linker was disordered in our structure, however it appeared to run towards the 5’ end of the RNA transcript, in a similar manner to the bacterial σ finger (Figures 4A & 4C). Although the tip of the GP68 linker was partially disordered, we found that is rich in negatively-charged residues (288-DKGGIDDDD-296), which might contribute to accumulating structural stress when approaching the DNA, in the same manner as in eubacterial msRNAPs (Murakami et al., 2002).

### The GP68 NTD appears likely to cycle between dissociated and associated conformations as in bacteriophage AR9, sampling the locality for promoter DNA

The N-terminus of GP68 was not resolved to sufficiently high-resolution in the ΦKZ nvRNAP-p119L RNA/DNA template structure to allow it to be modelled *de novo*, however we were able to dock and validate an accurate structural model generated with Alphafold2 (Jumper et al., 2021) to secondary structural resolution. According to our structural model, the GP68 NTD is located in the same position within the complex as region 2 of other bacterial σ^70^-like factors. Moreover, the N-terminus of GP68 would interact with the two α-helices of the clamp, which are a binding surface for σ factors in eubacterial msRNAPs (Lane & Darst, 2010a; 2010b). Region 2 of bacterial σ factors specifically interacts with the -10 element of bacterial promoters, hence participating in promoter recognition and stabilisation of the open promoter complex. Considering both the structural model and the position within the complex of GP68, we suspect that the GP68 NTD is involved in promoter recognition and transcription bubble formation. Although the topology of the GP68 NTD is quite divergent from that of bacterial σ factors, the electrostatic potential surface of that domain supported a role in DNA binding (Figures 4B & 4D). The promoter recognition subunit is also structurally similar in the ΦKZ and the AR9 nvRNAPs. Both subunits are positioned in the same location within the complex and display the same domain organisation, and non-template-strand single stranded DNA binds this subunit in the AR9 nvRNAP structure. The GP68 linker and the NTD were completely disordered in the nvRNAP holoenzyme structure without DNA and, although the resolution was substantially improved, the NTD was only resolved to intermediate resolution in the ΦKZ nvRNAP structure bound to the RNA/DNA p119L-template, which suggested that it is probably a mobile element. Previous limited proteolysis results showed that full-length GP68 was protected from proteolysis in the presence of DNA and that such protection was stronger with longer dsDNAs (de Martín Garrido, Orekhova, et al., 2021). Similarly to our observations in the ΦKZ nvRNAP, the GP226 NTD was also unresolved in the AR9 nvRNAP structures in the absence of DNA. All the evidence strongly supports a model in which the GP68 linker and NTD would transition from an open state, further from the nvRNAP core to a closed conformation during promoter recognition, playing a crucial role in promoter recruitment, bubble formation, and bubble stabilisation. The consensus sequence of promoters recognised by AR9 nvRNAP is located between positions -11 and -8 relative to the transcription start site, while the consensus sequence of late promoters recognised by the ΦKZ nvRNAP overlaps with the transcription start site ((-3)-TATG-(+1)). Moreover, unlike ΦKZ, the AR9 genome contains uracils in the template strand. Fraser and colleagues showed that the AR9 promoter-recognition subunit recognises an A-rich motif of the non-template strand upstream of the consensus region and forms a specific pocket for the U-rich consensus region together with the β-like subunit GP105, providing a specific mechanism for promoter recognition and bubble formation (Fraser et al., 2022). As our map exhibits low local resolution in the GP68 NTD region, including for the non-template DNA strand of the transcription bubble, it remains unclear whether or not this subunit specifically recognises ΦKZ late promoters, or if further factors are required for bubble formation, however it is notable that in the cell ΦKZ nvRNAP works in a complex environment due to the presence of the bacteriophage nucleus (Antonova, Belousova et al., 2023), whereas bacteriophage AR9 does not form a nucleus-like structure inside the cell.

### Bacteriophage AR9 and ΦKZ nvRNAPs appear to share common conserved core transcriptional mechanisms, but must have divergent promoter recognition mechanisms

The high level of similarity between the four β/β’-like subunits of the AR9 and ΦKZ nvRNAP, compared to the greater degree of divergence between their promoter recognition subunits both in sequence and, to a lesser extent, structure, is in line with our hypothesis of the σ subunit being transferred initially to an ancestral bacteriophage genome and being mutated to favour viral promoter recognition, hence becoming highly diverged from bacterial σ factor. Genes encoding the β/β’-like subunits would have been transferred later, thus explaining the lesser degree of their divergence with respect to their eubacterial homologues. Comparison of the structural features of AR9, *E. coli*, and ΦKZ RNAP nucleotide clefts (Supplementary Table 2) implies that the vast majority of the mechanism of transcription elongation, rather than promoter recognition and associated mechanisms, remains conserved in these deviant, split-subunit, msRNAPs. There are several key differences from canonical msRNAPs. however; most noticeably the missing rudder, implying that there may be less robust or different stabilisation of the transcription bubble in such enzymes. Our structure of a transcribing complex provides concrete evidence that this expected conservation of the mechanism of transcription elongation is the case. The addition of nucleic acids also revealed the structure of novel domains that were weakly-resolved in the ΦKZ nvRNAP apo-structure (de Martín Garrido, Orekhova, et al., 2021), including the clamp, the GP74 trigger loop insertion and the GP123 middle domain, equivalent to the bacterial β2 domain. All these regions are probably involved, to some extent, in the stabilisation of the open promoter complex. We therefore suggest that they probably transition between different conformations, adopting a closed conformation, being closer to the core of the enzyme, that stabilises the transcription bubble in the presence of DNA. Overall, we note that all the main differences between the bacteriophage AR9 and ΦKZ nvRNAPs lie in regions key for promoter recognition; the N-terminal β’ Zn^2+^ binding domain is missing in AR9 but not in ΦKZ, there is a second putative DNA-binding interface in the GP68 CTD corresponding to σ region 4 in comparison to AR9, and there is very low similarity between the sequences and positions of the consensus promoters recognised by the ΦKZ nvRNAP. Given the dependence of the AR9 nvRNAP promoter recognition on uracil, and the requirement for bacteriophage nuclear localization of the ΦKZ nvRNAP for transcription inside the cell, we suspect that the promoter recognition mechanisms of the ΦKZ and AR9 nvRNAPs must be divergent, and postulate that further factors or regions yet to be uncovered are required for ΦKZ nvRNAP transcription initiation *in vivo*.

## Materials and Methods

### Cloning

A set of pET-based plasmids containing each of the five ΦKZ nvRNAP subunit genes, and a plasmid (pnvCo-Ex) containing the four β/β’-like subunits, were generated previously to express recombinant ΦKZ nvRNAP that is functionally equivalent to the native polymerase and can be obtained in larger amounts with a less laborious protocol (Orekhova et al., 2019). Briefly, the individual ΦKZ nvRNAP genes, GP55, GP74, GP71-73 and GP123, were cloned into a set of pET-based plasmids and later assembled in a single pnvCo-Ex plasmid by blunt ended insertion of PCR-fragments containing the T7 promoter, lac operator, RBS and one of the genes of the ΦKZ nvRNAP from each of the pET-set of plasmids, into pACYC184 (Orekhova et al., 2019). The pGp68 plasmid was also previously produced by assembly of two PCR-fragments using the NEBuilder HiFi DNA Assembly Master Mix (New England Biolabs, Ipswich MA, USA) according to the manufacturer’s protocol as detailed in (Orekhova et al., 2019). Combination of both these vectors within BL21(DE3) *E. coli* allowed co-expression of the complete ΦKZ nvRNAP complex.

### Protein expression and purification

Protein was expressed and purified as reported in de Martín Garrido et al., 2021. BL21(DE3) *E. coli* cells were transformed with two plasmids (pGp68 and pnvCo-Ex) with compatible origins to express the full ΦKZ nvRNAP complex. Expression was induced by the addition of 1 mM IPTG at an OD600 of 0.5-0.7 and cells were then incubated at 22 °C for 3 h. Recombinant ΦKZ nvRNAP complexes were purified as previously described (Orekhova et al., 2019). Briefly, 1 g wet mass of cells was disrupted by sonication in 10 mL buffer A (40 mM Tris-Cl pH 8.0, 10% (v/v) glycerol, 500 mM NaCl, 1 mM DTT) containing 5 mM imidazole followed by centrifugation at 11 000 *rcf* for 30 min at 4 °C. The soluble fraction was loaded onto a HisTrap HP 1 mL (GE Healthcare Life Sciences, now Cytiva, USA) previously equilibrated, and then washed with buffer A supplemented with 5 mM imidazole. Recombinant complexes were then eluted with buffer A supplemented with 250 mM imidazole. Size-exclusion chromatography was performed over a Superdex 200 Increase 10/300 GL (GE Healthcare Life Sciences, now Cytiva, USA) in TGED buffer (20 mM Tris-Cl pH 8.0, 5% (v/v) glycerol, 0.5 mM EDTA, 1 mM DTT, 200 mM NaCl). Fractions containing ΦKZ nvRNAP were concentrated to 1 mg/mL (Amicon Ultra-4 Centrifugal Filter Unit with Ultracel-30 membrane, EMD Millipore, Merck, USA) and then stored flash frozen at -80 °C until grid preparation.

### DNA/RNA-template preparation

The p119L RNA-DNA template used for cryo-EM was prepared from oligonucleotides ordered through Integrated DNA technologies (IDT). The DNA sequences were AGTAATTTTAGTGAATGTATTTGCTATA TTGCTATCCTAACAGTTCCCAAAAGCCTAAAGTTACAATATAGGTAC and GTACCTATATTGTAACTTTAGG CTTTTGGGAACTGTCTACATAGCAATATAGCAAATACATTCACTAAAATTACT, while the priming RNA sequence was Cy5-GUAGA (Figure 1a). DNA and RNA/DNA templates were prepared by mixing an equal volume of each oligonucleotide at a final concentration of 1 μM, incubated at 95 °C for 5 min and cooled to 30 °C by increments of -1.1 °C/min. After that, annealed oligonucleotides were left on ice for 10 min and kept at -20 °C until use. To check that oligonucleotides were properly annealed they were analysed on native PAGE and stained with SYBR Safe (Invitrogen, Thermo Fisher Scientific, USA).

### *In vitro* transcription

Thawed ΦKZ nvRNAP at 0.1 mg/mL final concentration was mixed with the p119L RNA/DNA scaffold at a 1:1 ratio in transcription buffer (40 mM Tris-Cl pH 8.0, 10 mM MgCl_2_, 5 mM DTT) in the presence of 1 mM ATP, CTP, and GTP. UTP was withheld in order to force transcription to halt after three nucleotides. The resulting reactions were incubated for 30 min at 37 °C to allow binding and extension.

### Native PAGE Gel-shift Assay

Native PAGE was performed to confirm ΦKZ nvRNAP binding to the p119L RNA/DNA template using 4–20% Mini-PROTEAN TGX Precast Protein Gels (Bio-Rad Laboratories, Inc., USA) (Supp. Fig. 1). Samples were prepared by mixing 10 μL of each sample with 10 μL of 2X Loading Buffer without SDS (63.5 mM Tris-Cl pH 6.8 and 40% Glycerol). Loading Buffer was prepared without any tracking dye to avoid masking the signal of Cy5-oligonucleotides. Hence, one well was loaded with 4x LDS Sample Buffer (Expedeon, now Abcam, UK) to monitor the front of the gel. 20 μL of each sample was loaded onto the gel, which was run for 40 min at 120 V at 4 °C. To visualise Cy5-RNA, gels were imaged without staining, while to visualise DNA, gels were stained with SYBR Safe (Invitrogen, Thermo Fisher Scientific, USA). Gels were imaged on a Gel Doc XR+ (Bio-Rad Laboratories, Inc., USA) using the pre-set protocols for the Cy5 channel, SYBR Safe, and Coomassie stain as appropriate.

### Grid preparation

Holey carbon copper grids (Quantifoil R 2/1 on 300 copper mesh) (Quantifoil, Germany) were washed with ultrapure water and ethyl acetate (Sigma-Aldrich, USA) to eliminate any residual contamination from the grid production process. After washing, grids were left to air-dry. The washed grids were then plasma cleaned using a Basic Plasma Cleaner PDV-23G-2 (Harrick Plasma, USA) for 10-15 s in air using the high mode setting. Addition of graphene oxide films was carried out according to the published protocol (de Martín Garrido, Ramlaul, et al., 2021). A 1% (w/v) graphene oxide and 0.01% (w/v) DDM solution was prepared by mixing 2.5 μL of 2 mg/mL graphene oxide (Sigma-Aldrich, USA), 2.5 μL of 1% DDM (GLYCON Biochemicals GmbH, Germany) and 245 μL of ultrapure water (final volume 250 μL). Immediately before use, the mixture was vortexed for 1 min to ensure that the graphene oxide was properly suspended. Drops of 5 μL of graphene oxide – DDM suspension and 5 μL of ultrapure water were prepared on the clean face of a piece of parafilm. Firstly, a grid was gently deposited on top of the suspension droplet and incubated for 1 min with the carbon-covered side in contact with the liquid. After incubation, the grid was lifted with negative-action tweezers and the droplet of water subsequently recovered by gently touching the liquid gently with the copper side of the grid. At this point, while holding the grid with the tweezers, there should be a droplet of suspension on the carbon-covered side of the grid and a droplet of water on the copper side. Each grid was then carefully blotted with the water droplet-side down against a piece of filter paper.

### Cryo-EM sample preparation

Cryo-EM samples for the ΦKZ nvRNAP complexes bound to the p119L RNA/DNA template were prepared immediately after the transcription reaction. After incubation, ΦKZ nvRNAP complexes bound the p119L RNA/DNA template were adsorbed to the thin film of graphene oxide deposited upon the surface of grids prepared as above. After application of sample to the grids, samples were plunge-frozen in liquid ethane using a Vitrobot Mark IV (Thermo Fisher Scientific, USA) at 4 °C and 100% humidity, -6 blot force, 4 s waiting time and 0.5-1 s blotting. The blotting pads were prepared with Whatman number 41 filter paper 55 mm discs (GE Healthcare Life Sciences, now Cytiva, USA).

### Screening of vitrification conditions

ΦKZ nvRNAP-p119L RNA/DNA template cryo-grids were clipped into autoloader clip-rings (Thermo Fisher Scientific, USA) and screened for distribution on a Glacios cryo-TEM (Thermo Fisher Scientific, USA) equipped with a Falcon 4 direct electron detector located at the EM facility at the Centre for Structural Biology at Imperial College London. The microscope was operated at 200 kV, a magnification of 150 000 fold, and over an applied defocus range of between -1.2 and -2.5 μm.

### High-resolution Data Collection

The selected, screened grid was recovered from the Glacios and transferred to a Titan Krios G3i equipped with a Falcon 4i direct electron detector and a Selectris Filter (Thermo Fisher Scientific, USA) at the Electron Bio-Imaging Centre (eBIC) at Diamond Light Source. The microscope was operated at 300 kV, at a magnification of 150 000 fold and over an applied defocus range of -1.4 to -2.9 μm, collecting movie images with a total exposure of 51 e^-^/Å^2^. A total of 3 092 movies were recorded automatically using EPU with an object pixel size of 0.92 Å/pixel (Supp. Figure 2; Supp. Table 1).

### Image Processing

Frames were aligned and dose-weighted with MotionCor2 (Zheng et al., 2017) and CTF parameters were estimated with CTFFIND4 (Rohou & Grigorieff, 2015). Micrographs displaying irregularities in their Thon rings and those that did not contain high-resolution information according to power spectrum Thon ring fitting were discarded, leaving 2 545 micrographs for further refinement (Supp. Figure 2). 1 198 587 particles were selected semi-automatically using BATCHBOXER (Tang et al., 2007) and were classified into 2D class averages with RELION 4 (Kimanius et al., 2021) leaving CTF information up to the first peak intact. Particles yielding high-resolution class averages (265 839) were retained for further refinement. This set of particles was further classified into six 3D classes without alignment using a high tau fudge value of 16. One of the classes containing 83 389 particles had better defined density for the DNA and peripheral regions of the complex. CTF and aberration refinement (including beam-tilt, anisotropic magnification, and per-particle CTF estimation) and Bayesian polishing using RELION 4 (Kimanius et al., 2021) were performed on this set of particles, which finally led to a 3.3 Å reconstruction, according to an independent half-set FSC of 0.143, using the auto-refinement procedure in RELION 4 (Supp. Figure 3). Particles contributing to this high-resolution reconstruction were further refined to improve density in the catalytic core, DNA and GP68 NTD regions. 3D classification with a fine angular search (--sigma_ang of 2.0) yielded a class with well-defined DNA at the downstream region and the catalytic core, which included 39 919 particles. Those particles contributed to a final high-resolution reconstruction using the auto-refinement procedure in RELION 4 which reached 3.3 Å resolution according to an independent half-set FSC of 0.143 (Supp. Figures 3 & 4).

### Modelling and refinement

Structural models for the uncompleted regions of the ΦKZ nvRNAP structure (7OGP) (de Martín Garrido, Orekhova, et al., 2021) were generated using AlphaFold2 (Jumper et al., 2021). These models corresponded to the middle domain of GP74 (367-545), middle domain of GP123 (143-337), GP55-clamp (1-291) and N-terminus of GP68 (1-318). Molecular models were then rebuilt with COOT (Emsley & Cowtan, 2004; Emsley et al., 2010) and expanded from the well-defined regions, as most of the secondary structure elements were already in agreement with the map. The density for the GP68 N-terminal region was not resolved at high-resolution in the 3.3 Å reconstruction, but the Alphafold2 structural model could be readily docked into the filtered density (Figure 1). Within the catalytic core, the RNA hybridised along the template ssDNA strand was built in COOT (Emsley & Cowtan, 2004; Emsley et al., 2010) according to the expected RNA sequence. The downstream dsDNA was well-defined in our reconstruction and, based on the RNA sequence, sequence for the template dsDNA strand was assigned at the catalytic core, and expanded to the downstream dsDNA away from the transcription start site. The ssDNA strand forming the transcription bubble was visible up to position - 3 relative to the transcription start site, whereas the non-template ssDNA strand was not visible. The connection of the template strand forming the transcription bubble with the upstream dsDNA was not well-resolved in our reconstruction. The upstream dsDNA region was lower resolution than the downstream dsDNA, however at low contours the phosphate backbone was visible and a 17 bp dsDNA structural model with unassigned sequence was built in COOT (Emsley & Cowtan, 2004; Emsley et al., 2010) (Figure 1). The atomic model was refined with PHENIX real-space refine (Liebschner et al., 2019). A summary of the refinement statistics can be found in supplementary table 1 including the B-factor calculated with B-average (CCP4). Homology searches and Cα comparisons were carried out using the DALI-lite server (Holm & Sander, 1995), while surface area calculations were performed using the PISA server.

## Supplementary Material

Supplementary Materials

## Data Availability

The cryo-EM density map resolved for the ΦKZ nvRNAP-p119L-RNA complex has been deposited in the EM Databank under accession code EMD-18661, while the corresponding molecular model has been deposited in the protein data bank as PDB-ID 8QUE.
