# Supplementary material for: Structure of the Bacteriophage PhiKZ non-virion RNA Polymerase transcribing from its promoter p119L
Source: J Mol Biol. Author manuscript; Available in PMC 2025 Dec 9. (PMC7618050; doi:10.1016/j.jmb.2024.168713)
Supplement: Supplementary Materials [file EMS207956-supplement-Supplementary_Materials.pdf]

Supplementary Information

Structure of the Bacteriophage PhiKZ non-virion RNA Polymerase transcribing from its promoter p119L

Natàlia de Martín Garrido[1], Chao-Sheng Chen[1], Kailash Ramlaul[1], Christopher H. S. Aylett[1]†, and Maria Yakunina[2]†

[1] Section for Structural and Synthetic Biology, Department of Infectious Disease, Imperial College London, London, United Kingdom.
[2] Shenzhen MSU-BIT University, 1 International University Park Road, Dayun New Town, Longgang District, Shenzhen, Guangdong Province, People's Republic of China, 518172.

† To whom correspondence may be addressed:

CHSA c.aylett@imperial.ac.uk
MY yakuninam@gmail.com

Keywords: β subunit; RNA polymerase; β′ subunit; Cryo-EM; Jumbo-phage; σ-factor; Single-particle analysis, phiKZ.

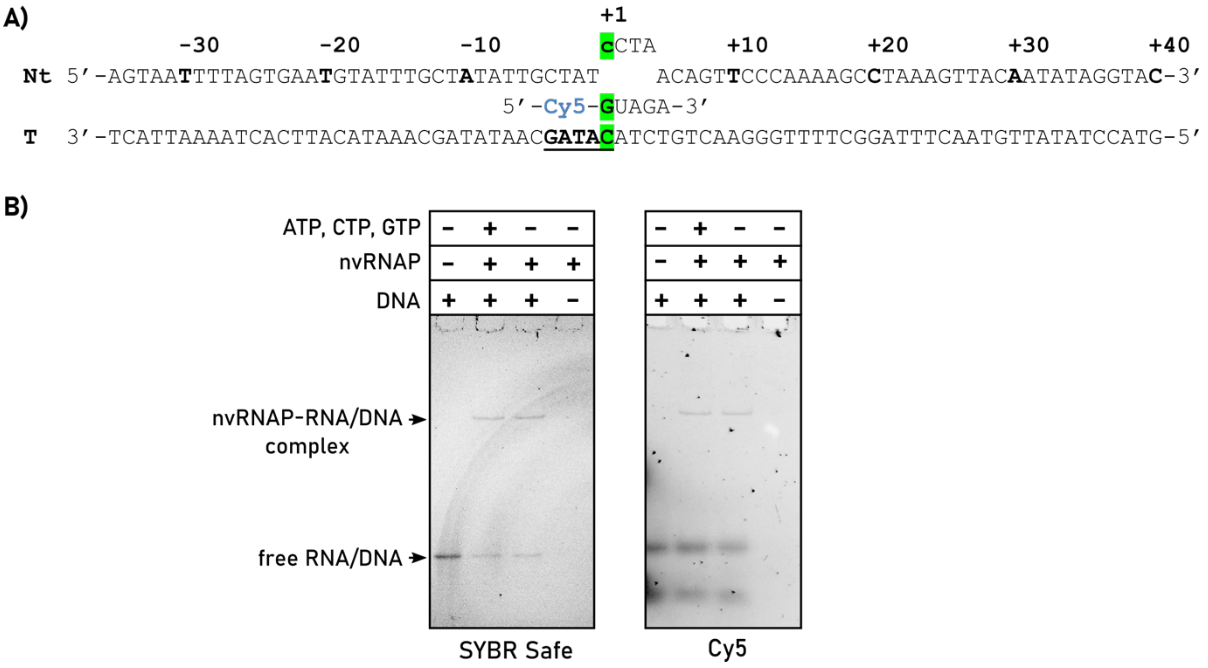

Supplementary Figure 1: Analysis of nvRNAP binding to p119L-4nt RNA/DNA template. (A) The form and sequence of the p119L-4nt RNA/DNA template. The transcription start site is coloured green and the short consensus region at the start site is highlighted in bold and underlined. (B) Analysis of the binding of the nvRNAP to the designed RNA/DNA template by gel shift. 4–20 % Mini-PROTEAN TGX Precast Protein Gel (Bio-Rad Laboratories, USA) stained with SYBR-Safe (left) and imaged in the Cy5 channel (right). Samples run in each lane of the gel are indicated at the top of each gel image.

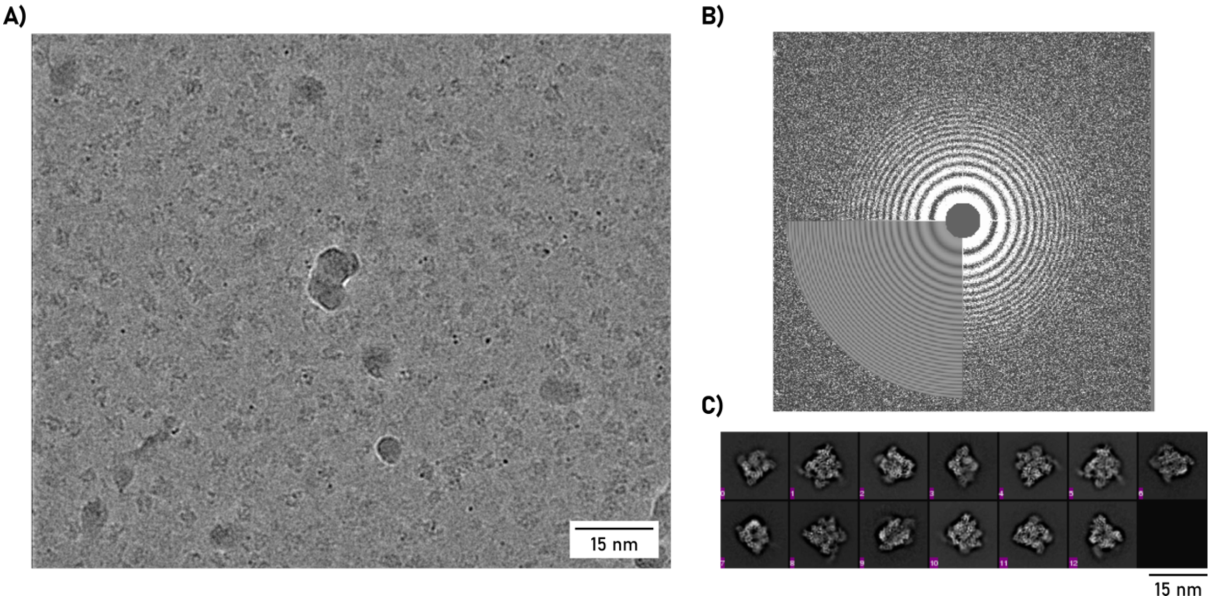

Supplementary Figure 2: High-resolution data collection of the ΦKZ nvRNAP in complex with an RNA/DNA p119L template. (A) Representative micrograph of nvRNAP complex bound to p119L RNA/DNA scaffold adsorbed to a graphene oxide film deposited onto a copper grid. (B) Thon rings simulated with the estimated CTF parameters (bottom left) and experimental power spectrum fitted. (C) Representative 2D class averages representing different views of the ΦKZ nvRNAP in complex with an RNA/DNA p119L template.

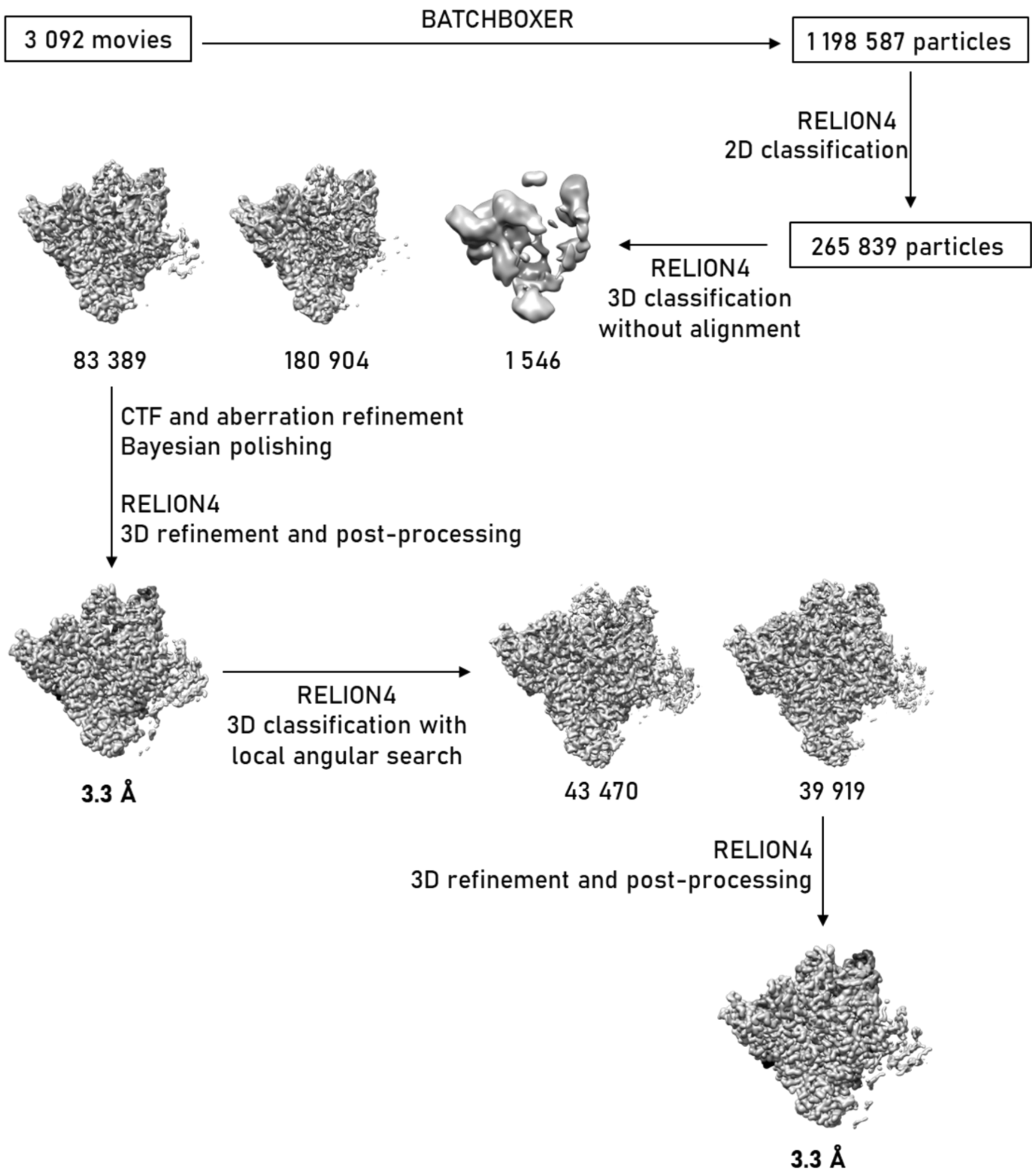

Supplementary Figure 3: Data processing scheme for the high-resolution cryo-EM data collection of the ΦKZ nvRNAP complex bound to the p119L RNA/DNA template. Flow-chart depicting the data processing procedure. Particle numbers retained at each stage of the process are indicated below each 3D reconstruction.

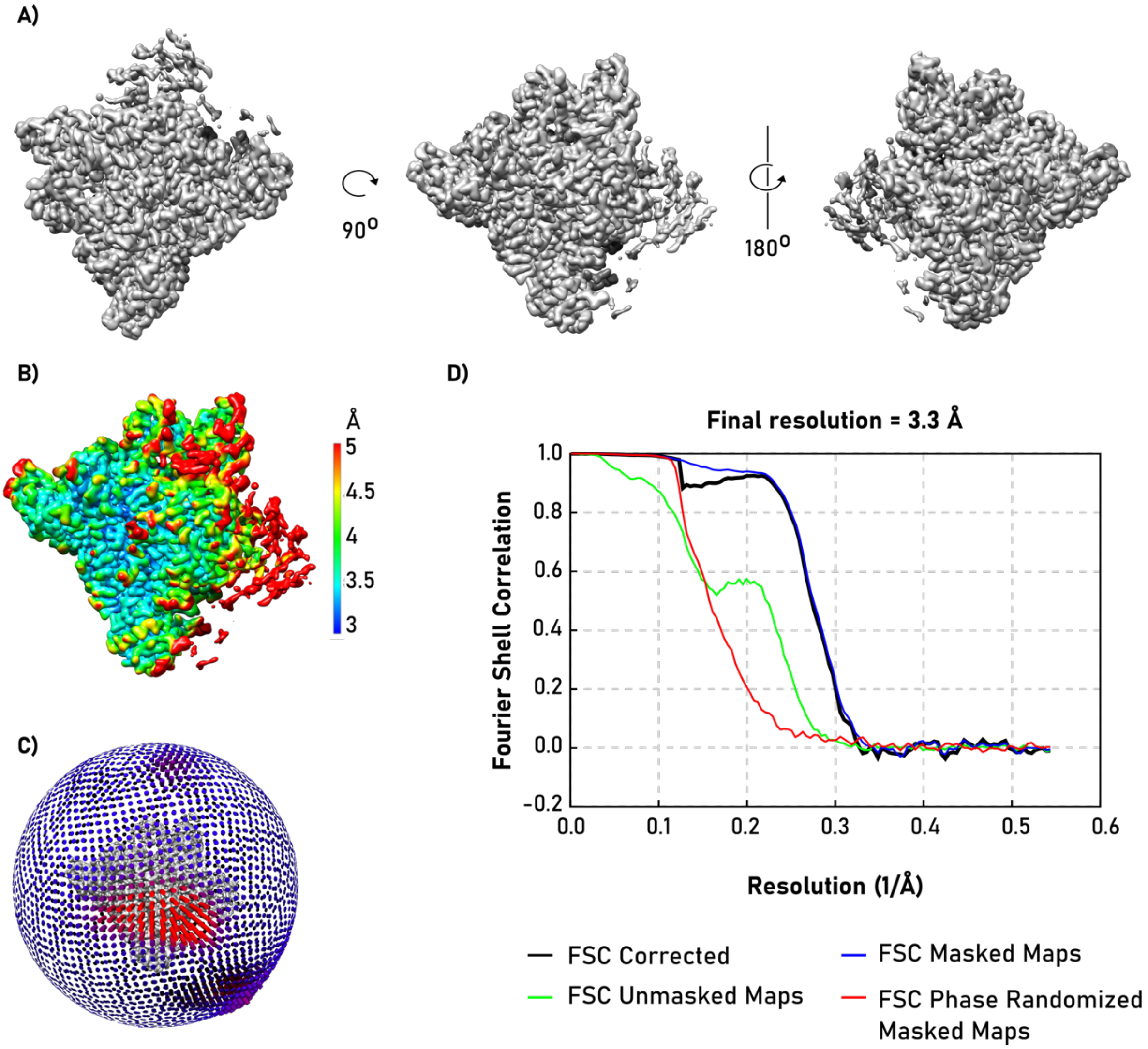

Supplementary Figure 4: High-resolution 3D reconstruction of the ΦKZ nvRNAP bound to the p119L RNA/DNA template. (A) 3.3 Å reconstruction of the ΦKZ nvRNAP bound to the p119L DNA/RNA template in different views. (B) Local resolution map of the 3.3 Å reconstruction calculated using BLOCRES. (C) Angular distribution plot of the 3.3 Å reconstruction. D) Global FSC curve for the 3.3 Å reconstruction.

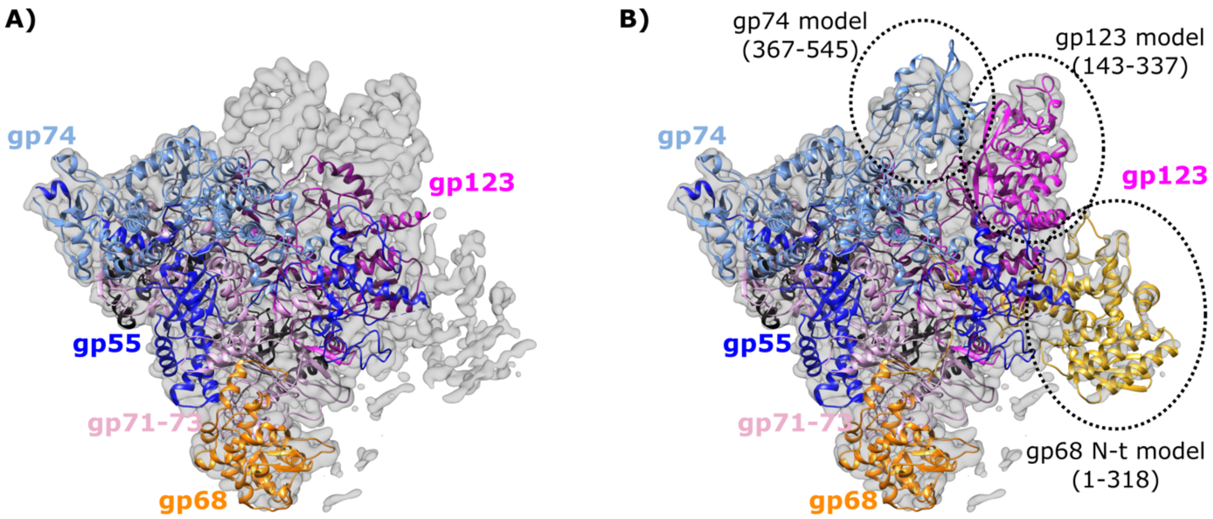

Supplementary Figure 5: New features of the high-resolution 3D reconstruction of the ΦKZ nvRNAP bound to the P119L RNA/DNA template. (A) The nvRNAP structure without DNA (PDB 7OGP) fitted into the cryo-EM map that yielded the current ΦKZ nvRNAP complex bound to the P119L RNA/DNA template. (B) The structure of the ΦKZ nvRNAP complex bound to the P119L RNA/DNA template. Models for gp74 middle domain (396-533), gp123 middle domain (146-336), and gp68 N-terminus and linker (1-273) can be fitted into the extra densities.

```
E.coli_beta'      MKDLLKFLKAQTKTEEFDAIKIALASPDMIRSWSFGEVKKPETINYRTFKPERDGLFCAR      60
PHIKZ_gp55        MGLYAKVVDHNEVHDQFTGKRIYA--ND------Y---NTSNSDE--KEEF------DRH      41
AR9_gp270         MGKKLSLIDFNEIYNE---ENLI---------------TRANPIE--NHEFSDDGIYSER      40

E.coli_beta'      IFGPVKD-------YECLCGKYKRLKHRGVICEKCGVEVTQTKVRRER-MGHIEL-----      107
PHIKZ_gp55        FYSHFQDSEAIESSVSCDCRAIEDAHKLGVICDICNTPVVNTSSRPIEPSMWVRTPKHVR      101
AR9_gp270         IFGSYNE---------------------------------DDDDKDIDTIGWINIEP--Y      65

E.coli_beta'      --ASPTAHIWFLKSLPSRIGLLLDMPLRDIERVLYFESYVVIEGGMTNLERQQILTEEQY      165
PHIKZ_gp55        SLINPRLIIMLTGYLVTKEFDFLAYL----TDTSYRYDVESIGSKETRRKVDRLL-HRGF      156
AR9_gp270         YIINPILFTIIKKCIPS-INKIIN----------YQQSIDQNGE---NIDLTEEI-GEDD      110
```

Supplementary Figure 6: Sequence alignment of the *E.coli* β’ subunit, the ΦKZ nvRNAP subunit gp55 and the AR9 nvRNAP subunit gp270 . Cysteine residues forming the Zn binding domain at the N-terminus of the bacterial β’ subunit and ΦKZ gp55 are highlighted in green.

Magnification (×)	150 000
Voltage (kV)	300
Electron exposure (e^{-}/Å^2)	51
Defocus range (µm)	-1.4 to -2.9
Pixel size (Å/pix)	0.92
Symmetry imposed	C1
Initial number of particles	1 198 587
Final number of particles	39 919
Map resolution (Å) at FSC = 0.143	3.3
Model-Map CC Mask / Model-Map CC Volume / Mean B-factor	0.8154 / 0.7799 / 43.046
Molprobity overall score / clash score	1.57 / 4.51
RMS deviations in bond lengths (Å) / bond angles (°)	0.003 / 0.694
Ramachandran favoured / allowed / disallowed (%)	95.06 / 4.90 / 0.04
Rotamers favoured / allowed / disallowed (%)	95.05 / 4.64 / 0.31

Supplementary Table 1: Collection parameters and model refinement statistics for the cryo-EM structure of the ΦKZ nvRNAP bound to the p119L RNA/DNA promoter. Data were acquired on a Titan Krios G3i cryo-TEM (Thermo Fisher Scientific, USA) equipped with a Selectris energy filter and Falcon 4i direct electron detector located at the Electron Bio-Imaging Centre at Diamond light source.

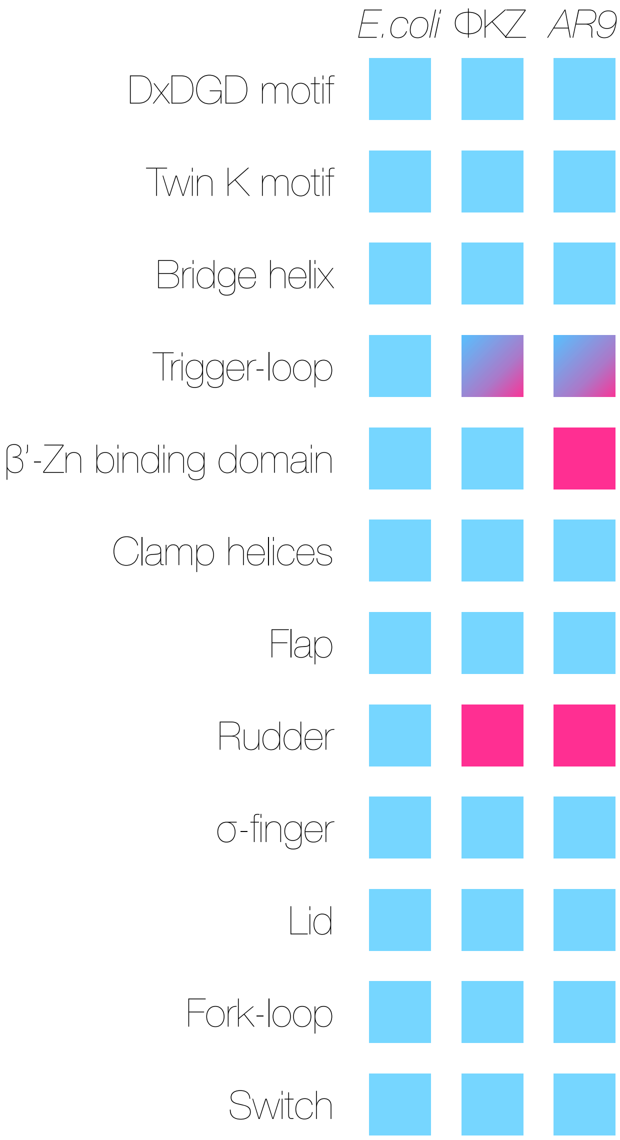

Supplementary Table 2: Conservation of the key elements of the nucleotide binding clefts of the *E. coli* msRNAP, ΦKZ nvRNAP, and AR9 nvRNAP. Schema showing the presence or absence of the key elements of the cleft stabilising the transcription bubble and catalysing RNA polymerisation. Blue indicates conservation from canonical RNAPs / presence, whereas pink indicates absence. Mixed colours indicate partial conservation with novel features / domains.
